# Changes in Selection Regime Cause Loss of Phenotypic Plasticity in Planktonic Freshwater Copepods

**DOI:** 10.1371/journal.pone.0090010

**Published:** 2014-02-26

**Authors:** Sergej Vital’evič Sereda, Thomas Wilke, Roland Schultheiß

**Affiliations:** 1 Department of Animal Ecology and Systematics, Justus Liebig University Giessen, Giessen, Germany; 2 Department of Biology, University of Turku, Turku, Finland; Fred Hutchinson Cancer Research Center, United States of America

## Abstract

Rapid phenotypic adaptation is critical for populations facing environmental changes and can be facilitated by phenotypic plasticity in the selected traits. Whereas recurrent environmental fluctuations can favour the maintenance or de novo evolution of plasticity, strong selection is hypothesized to decrease plasticity or even fix the trait (genetic assimilation). Despite advances in the theoretical understanding of the impact of plasticity on diversification processes, comparatively little empirical data of populations undergoing diversification mediated by plasticity are available. Here we use the planktonic freshwater copepod *Acanthodiaptomus denticornis* from two lakes as model system to study UV stress responses of two phenotypically different populations under laboratory conditions. Our study reveals heritable lake- and sex-specific differences of behaviour, physiological plasticity, and mortality. We discuss specific selective scenarios causing these differences and argue that phenotypic plasticity will be higher when selection pressure is moderate, but will decrease or even be lost under stronger pressure.

## Introduction

Population divergence can be driven by disruptive selection or–if migration is absent–by genetic drift. The former process constitutes a case of adaptation to different fitness optima and can be facilitated by phenotypic plasticity (PP) in the selected trait [1]. Subsequently, the phenotypes may become fixed and gene flow between populations will cease due to selection against (maladapted) migrants [2]. This process can lead to the adaptive evolution of species with fixed traits, derived from a common plastic ancestral population. This ‘flexible stem’ model of evolution [3] has been proposed to explain the formation of species flocks with similar ecomorphs, for example, within African cichlids [4] or threespine sticklebacks [5].

It is in generally believed that recurrent environmental fluctuations (i.e., following reliable cues like annual day length changes) or migration between patches in a fine-grained environment favour de novo evolution of PP or its persistence [6]. The evolution of PP constitutes either a modulation of existing plasticity (genetic accommodation) or the fixation of an alternative phenotype (genetic assimilation or Baldwin effect) and may take place over surprisingly short time scales [7]. For example, in a seminal laboratory experiment the genetic assimilation of the bithorax-like phenotype in *Drosophila* was achieved within several months (twenty generations) [8]. Similarly, the genetic assimilation of a less melanised phenotype in a free-living *Daphnia* population required only a dozen generations under increased predation pressure [9]. Such rapid phenotypic adaptation is critical for populations facing rapid environmental changes in their native range or while invading new habitats. Lande [10] proposed a mechanism whereby the rapid evolution (i.e., increase) of PP would allow populations to conquer new adaptive peaks, thus facilitating persistence in a changing fitness landscape. Yet despite advances in the theoretical understanding of the impact of PP on diversification processes, there are still only few empirical studies of populations undergoing diversification mediated by PP [11]. This is partly due to the lack of suitable model systems.

Planktonic copepods constitute a prime model to study plasticity-mediated diversification processes. They are often exposed to harmful ultraviolet radiation (UV) [12] and encounter this threat either with diel vertical migration [13] or with a (plastic) accumulation of photo-protective compounds like mycosporine-like amino acids and carotenoids [14], [15]. The conspicuous red carotenoid pigmentation makes them, however, more vulnerable to visual predators, e.g. fish or salamanders [16]. Consequently, they reduce pigmentation rapidly in the presence of fish eluates, even under continued UV stress [17], [18]. In fact, carotenoid content was consistently higher in populations under low fish predation than in populations under high predation [19], [20]. Such differences were suggested to be accompanied by differences in phenotypic plasticity and, by extension, may increase their potential for phenotypic adaptation [6], [21].

**Figure 1:**
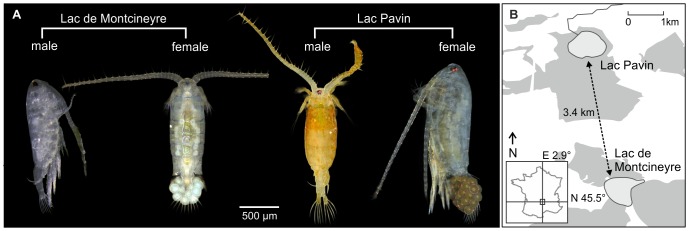
Study system. (A) Specimens of *Acanthodiaptomus denticornis* populations from the neighbouring lakes Lac Pavin (LP) and Lac de Montcineyre (LM). Both sexes in LM as well as LP females are translucent, whereas LP males bright red. (B) The lakes are situated in the French Massif Central. Grey areas around the lakes are woodland, white areas are mainly agricultural or other areas under human use.

The present study tests this hypothesized relationship between changes in phenotypic plasticity and selection regime. In particular, we investigated the impact of UV stress on two neighbouring populations of the freshwater copepod *Acanthodiaptomus denticornis* with differences in carotenoid pigmentation (Fig. 1). We found substantial population- and sex-specificity in physiological plasticity (i.e., changes in carotenoid pigmentation), UV-induced behaviour (i.e., vertical migration), and mortality. Our data indicates that the observed loss of ancestral phenotypic plasticity in one of the studied populations was directly caused by a change in selection regime in its natural habitat.

## Materials and Methods

### Ethics Statement

This study was carried out in strict accordance with the recommendations of the Convention of Biological Diversity (CBD). The required permits were issued by the Department of Economics, Evaluation and Integration of Sustainable Development (French Ministry for Ecology, Energy, Sustainable Development and Sea, Paris; Permit Number: SEEIDD-ERNR1-11-04-1925) and the Syndicat Intercommunal à Vocation Multiple de la Région d’Issoire de Communes de la Banlieue Sud Clermontoise (SIVOM, Coudes).

### Study System

The calanoid copepod *Acanthodiaptomus denticornis* (Wierzejski, 1887) is distributed mainly in the Alps, as well as in Scandinavian, French, and German low mountain ranges. The species is known for its characteristic bright red pigmentation of astaxanthin keto-carotenoids [22], described amongst others from Lac Pavin (hereafter LP) in the Massif Central in France. However, a translucent, unpigmented population was reported from Lac de Montcineyre (hereafter LM), a lake in close vicinity to LP ( [14]; Fig. 1). Both sub-alpine volcanic lakes have similar surface areas but differ in depth. LP has a maximal depth of 92 m and LM of 22 m [23], [24].

We collected specimens of *A. denticornis* in August 2011 from both lakes. Sampling was carried out from a boat with vertical plankton net hauls (mesh size: 150 µm; maximum haul depth: ∼15 m). The species was determined (a) morphologically based on the description in Einsle [25] and (b) genetically by blasting a fragment of the mitochondrial cytochrome c oxidase subunit I (COI) gene against NCBI GenBank entries (Mega BLAST algorithm). For the latter analysis, we isolated genomic DNA from four resting eggs from each population using the HotSHOT method [26]. COI amplification was carried out with universal primers [27] with slight modifications [28]; PCR conditions were as given in Schultheiß et al. [29]. Amplified fragments were sequenced in both directions by LGC Genomics (Berlin, Germany). Consensus sequence length was 659 base pairs.

### Culturing Conditions

Copepod cultures were started in August 2011 in polypropylene buckets (volume: 18 L) in climate chambers under constant temperature conditions (18°C) and a 14∶10 h light/dark cycle (Osram Lumilux de Lux 36 W/954 bulbs; light intensity 1900 lx on the culture ground level). We refer to this light henceforth as ‘white light’. The culturing medium (CM) contained 1 mL of 1 mol L^−1^ Tris-HCl per litre of re-mineralized distilled water (Re-Mineral Marine salt mix, Tropic Marin, Germany; pH value: 6.5–7.0). The medium was inoculated with two species of green algae that served as carotenoid source: *Chlamydomonas reinhardtii* (strain 77.81 provided by SAG, Culture Collection of Algae at Göttingen University, Germany) and *Scenedesmus acutus* (Division of Waste Management and Environmental Research, Giessen University). The copepod cultures were refreshed monthly by filtering the copepods through a 41 µm polyamide mesh and transferring them into fresh CM.

Non-axenic food cultures with strains of the ciliate *Paramecium caudatum* and the cryptophyte *Chilomonas paramecium* (‘Lebendkulturen – Schulbedarf’, Prien, Germany) were reared in CM, supplemented with 10 vol% of lettuce extract, 5 mg stigmasterol, and 5 wheat seeds per litre. Preliminary experiments indicated a daily food supply of 50 ciliates per copepod to be sufficient. We thus added appropriate volumes of food culture every 5 days to the copepod populations. Under these conditions, females produced both resting- and subitaneous egg clutches, which were released or hatched 2–4 days following egg extrusion. Within approximately 20 days, up to 30 nauplii per clutch developed into adults. Whereas these parameters attest to appropriate rearing conditions [30], [31], we also observed phases of prevailing resting egg production, male- or female-biased sex ratios, and excessive attachment of spermatophores to both sexes in all populations. Although these phenomena have been reported repeatedly for laboratory copepod cultures, they may also constitute properties of natural populations [32]–[34].

### Behavioural Response to UV Stress

#### Experimental setup

We were interested in differences of phototactic behaviour between the two lake population and studied lake- and sex-specific changes of vertical position in the water column following a UV cue. To exclude maternal effects, we started the experiment 12 generations after sampling the populations.

We set up 10 cylindrical 400 mL polycarbonate containers per lake population, each containing 5 males and 5 females in 350 mL CM enriched with ciliates. A lower and an upper compartment of the water column were defined by a horizontal line at the 150 mL mark; the total height of the water column was 11 cm. The 20 containers were placed randomly in line below UV tubes (Philips Actinic BL 15 W TL-D). UV-A radiation (315–400 nm) intensity was 7 W m^−2^ at the container ground level, measured with an ILT-XRD340B sensor mounted on a handheld radiometer IL1400A (International Light, USA). For reference: the UV intensity on a lightly clouded midday at the surface level of LM was 30 W m^−2^ (measured with the same radiometer).

During the experiment, the populations were exposed to UV light daily for three one-hour intervals (i.e., from 11∶00 h–12∶00 h, 13∶00 h–14∶00 h, and from 15∶00 h–16∶00 h). The number of male and female copepods in the two compartments of each container was recorded at the beginning of every UV light interval and after the interval under white light conditions. We thus obtained a total of six data points per day and container. The order in which the containers were documented was randomized for each recording. To further mitigate systematic errors, all recordings were carried out by the same investigator. The number of containers and animals to be studied enabled the recording of all containers within approximately 15 minutes. Two additional containers per lake population were not exposed to UV radiation and served as negative control (NC). Irrespective of the UV regime, we illuminated all containers with white light. Every 5 days the animals were transferred into clean containers with fresh food solution.

#### Statistical analyses

Differential responses to UV stress between populations and sexes were evaluated using a Bayesian logistic analysis of variance employing a binominal likelihood function and a beta prior. We simultaneously estimated eight predictor variables (β_i_) from our dataset (Table 1) and used a sigmoid link function to map the predictors to the observed dichotomous response (i.e., the position of an individual in the upper or lower compartment of the water column). Note that this model allows for an unbalanced experimental design [35], which was critical to our approach in light of sex- and lake-specific mortality rates (see below). In order to approximate the Bayesian posterior probability distributions of the predictors, we employed a Monte Carlo Markov Chain (MCMC) approach. MCMC samples were generated with a Gibbs sampler in R [36], [37] utilizing a modified protocol of Kruschke [35]. We ran a total of three chains with 50,000 generations each, a thinning of 1, and a burn-in of 500 generations. Preliminary runs were carried out to ensure that the length of the chains was sufficient for convergence.

**Table 1:** Lake- and sex-specific distributions of *A. denticornis* in the water column.

	Lac Pavin				Lac de Montcineyre			
	males		females		males		females	
	UV	WH	UV	WH	UV	WH	UV	WH
predictor	β_1_	β_2_	β_3_	β_4_	β_5_	β_6_	β_7_	β_8_
mean	1.18	−0.017	0.317	−1.36	0.15	0.588	−0.348	−0.506
95% HDI	0.836∶1.52	−0.274∶0.243	0.029∶0.602	−1.62: −1.11	−0.123∶0.417	0.29∶0.892	−0.6: −0.088	−0.765: −0.258

The table provides the posterior probability estimates of the employed predictor variables of a Bayesian logistic analysis of variance (β_i_; see text for details). The estimates indicate the deflection of each predictor from the overall central tendency and are the basis for the complex comparisons depicted in Fig. 3. Abbreviations: HDI–high density interval; WH–white light.

Behavioural differences were evaluated with the following sets of complex comparisons of the predictor variables: (1) distribution under UV light versus white light controlled for sex and lake population, and (2) male versus female distributions controlled for light regime and lake population. Differences are accepted as credibly nonzero if the area covered by the 95% high density interval (HDI) of the posterior probability distribution of a complex comparison excludes zero. As the form of a specific posterior distribution does not change with the number or structure of additional comparisons, the analysis is not prone to type I error inflation [35].

### Physiological Response to UV Stress

For the estimation of carotenoid concentrations, surviving animals were pooled after the experiment into batches of 3–12 individuals, separated by lake, sex, and treatment (i.e., UV exposed or negative control) and transferred into re-mineralized water for one hour to ensure gut evacuation. Subsequently, the animals were narcotized with soda water and egg sacs were removed. Carotenoid extraction in ethanol was carried out following the protocol of Hylander et al. [38]. Total carotenoid content per individual (C) was calculated as follows: C = (D×V×10^4^)×(E×W)^−1^, where D is the absorbance at 474 nm (astaxanthin), V is the volume of the extract in mL, E is the extinction coefficient for astaxanthin (2100), and W is the dry weight in mg [39]. We estimated dry weight from body length measured with a ColorView digital camera (Hamburg, Germany) mounted on a dissecting microscope, and the AnalySiS 5.0 software package (Soft Imaging System GmbH, Münster) using the length-weight relationship for con-generic species [40].

### Survival Analysis

We studied mortality in our populations by employing survival analyses. In particular, we were interested in the relationship between the survival distribution and three covariates, coded as follows: sex (males: 0, females: 1), lake (LM: 0, LP: 1), and treatment (NC: 0, UV exposed: 1). Lifetimes in our data were right-censored, that is, each death was recorded as event (1) and censoring stopped with the termination of the experiment (0). We used a Cox proportional-hazards regression model to fit our data with the covariates to the hazard function λ(t|X) = λ_0_(t)exp(δ’X), where λ_0_(t) is the baseline hazard, X the vector of covariates, and δ’ a vector of unknown parameters [41]. The parameters were estimated using partial likelihood functions. Additionally, we inferred sex-specific survival functions for each lake population based on the Kaplan-Meier estimate [42]. All calculations were carried out in R with the package ‘survival’ [43].

## Results

### Species Identity and Sexual Dimorphism

The COI sequences of eight specimens from both lakes were identical. Blasting against NCBI GenBank entries yielded 99% similarity between our sequences and a COI sequence of *A. denticornis* from Lake Piburg, Austria [44], thus confirming our morphological determination. The sequences were deposited in NCBI GenBank (accession numbers: KF360191– KF360198).

**Figure 2:**
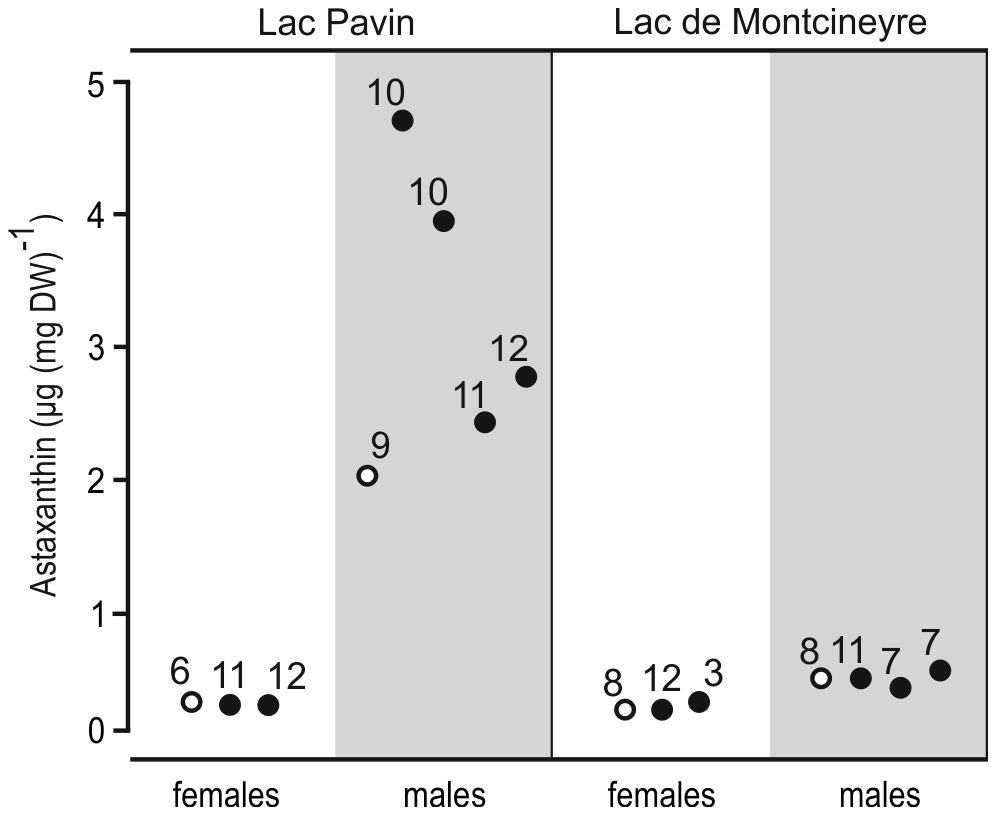
Changes of carotenoid content in *A. denticornis*. Empty circles refer to negative controls; black circles refer to UV exposed animals. Numbers above each point correspond to the number of animals in the respective batch of ethanol extract. A rise in astaxanthin content was only observed in LP males. In both populations males had higher concentrations of astaxanthin than females.

This clear taxonomic identification notwithstanding, we found a hitherto unreported sexual dimorphism in the LP population with brightly red coloured males but translucent females (Figs 1 and 2; see section ‘Physiological response to UV’ below). This observation was confirmed during our second visit to LP in May 2012. In contrast, both sexes in LM were translucent as previously reported in the literature.

### Behavioural Response to UV Stress

#### Distribution of specimens under white light

**Figure 3:**
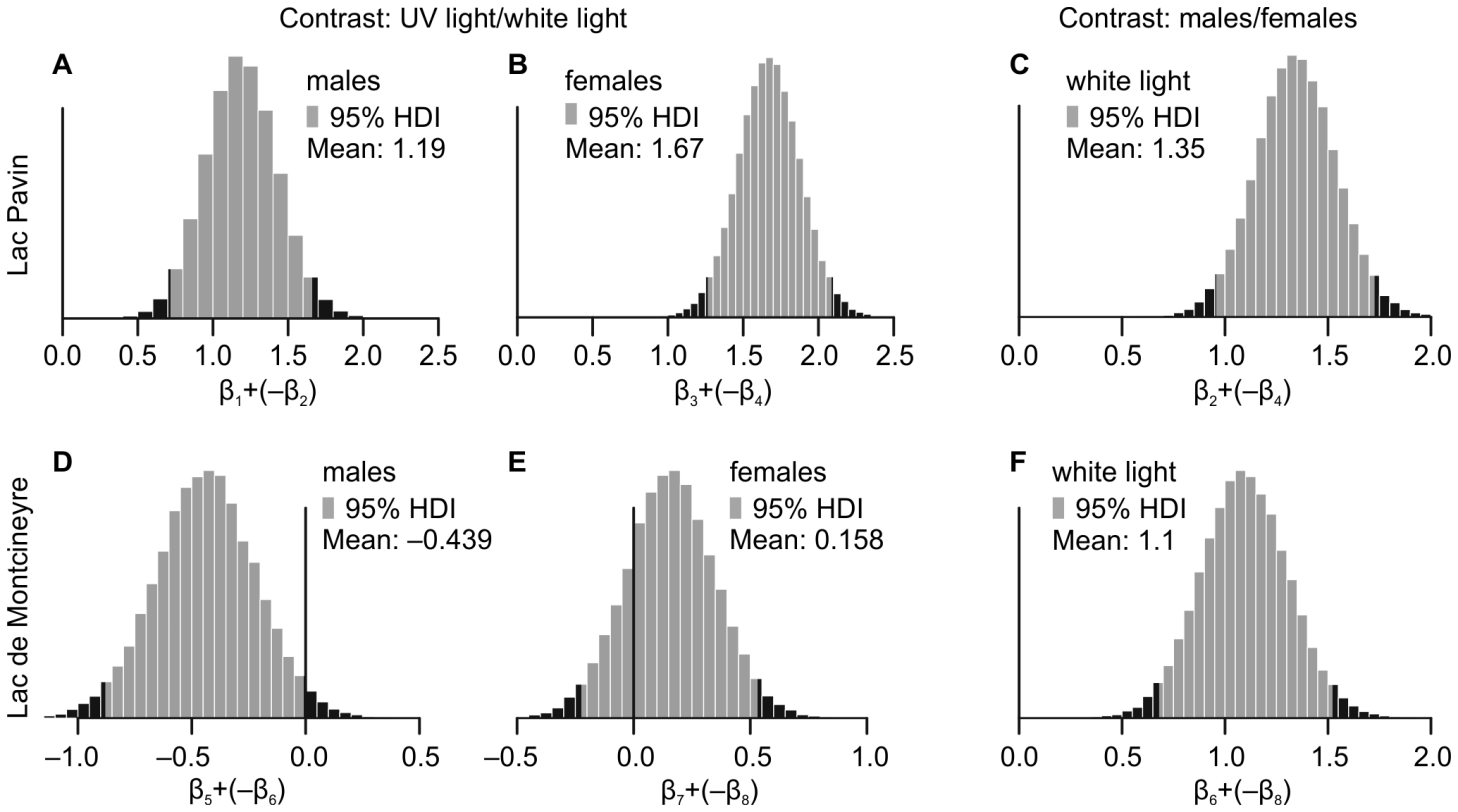
Bayesian contrasts of posterior probability estimates of the predictor variables in Table 1. High density intervals (95% HDI) are marked in light grey, values outside the intervals in dark grey. The y-axis represents the density of the probability distribution. Contrasts of distributions of UV versus white light are provided for both sexes of LP (A, B) and LM (D, E). Furthermore, we contrasted the distributions of males versus females under white light in LP (C) and LM (F).

Posterior probability estimates of the predictor variables indicated that LP males showed no preference for a particular compartment under white light (β_2_; all probability estimates are provided in Table 1). LM males, however, stayed mostly in the lower compartment (positive 95% HDI of β_6_), whereas females of both lakes preferred the upper compartments (negative 95% HDI of β_4_ and β_8_). Subsequent trials with two containers that contained only females showed, however, no preference of females for the upper compartment under white light. Contrasting male and female distributions revealed considerable sex-specificity in both lakes (Fig. 3C, F).

#### Distribution of specimens under UV light

A UV cue causes a change in the vertical distribution of LP copepods, resulting in a pronounced downward movement of both sexes as indicated by a shift of the 95% HDI estimates into the positive range (β_1_ and β_3_, Fig. 3A, B). In contrast, the distribution of LM females did not change (β_7_ in Table 1, zero included in 95% HDI of UV light/white light comparison in Fig. 3E). LM males exhibited a weak, yet not significant, tendency to move to the upper compartment of the water column (95% HDI of the comparison in Fig. 3D shifted almost completely into the negative range).

### Physiological Response to UV Stress

Females of both lakes possessed astaxanthin concentrations between 0.32 and 0.36 µg (mg W)^−1^ for UV-stressed and control specimens (Fig. 2). Astaxanthin content in LM males was almost twofold higher than in both female groups but did not change under UV stress (0.46−0.59 µg [mg W]^−1^). LP males on the other hand showed a considerable increase in carotenoid pigmentation after UV exposure. Their astaxanthin content ranged between 2.5 and 4.7 µg (mg W)^−1^ in the four UV exposed subsamples, and was 2 µg (mg W)^−1^ in the negative control.

### Survival Analysis

**Figure 4:**
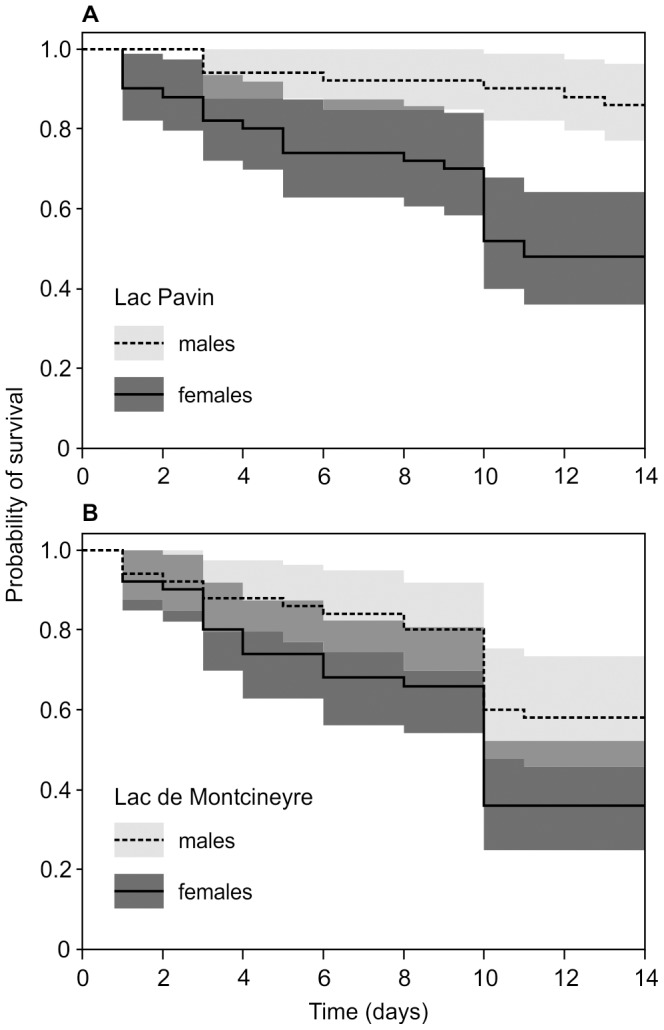
Sex- and lake-specific survival functions of *A. denticornis*. Probabilities of survival (based on Kaplan-Meier estimates) are plotted against experimental duration. Dashed lines are indicating males and straight lines females (shaded grey areas around each line represents the 95% confidence intervals of the respective survival function). Among the UV exposed populations, mortality was lowest for LP males and highest for LM females. More detailed statistics are provided in the text and in Table 2.

We evaluated the fit of the Cox model by testing the assumption of proportional hazards of the three covariates, all of which were not significant (Table 2). Hence the assumption is not violated. The examination of the Cox model revealed significant effects of the three covariates on the hazard (Table 2): The coefficient for ‘sex’ was negative, indicating that the expected hazard is higher in females than in males. Likewise, the negative coefficient for the covariate ‘lake’ suggested a higher hazard in LM. The ‘treatment’ coefficient was positive, indicating a higher hazard for UV exposed individuals. The overall null hypothesis that all δ estimates are zero was rejected (Likelihood ratio test = 35.59, Wald test = 31.63, logrank test = 33.77; degrees of freedom = 3; all p-values <0.001). The sex-specific survival curves for each lake based on Kaplan-Meier estimates are depicted in Figure 4.

**Table 2:** Effect of the covariates lake, sex, and treatment on survival.

	proportional hazards			effect of covariates		
	rho	chi-squared	p-value	Coef(δ)	Coef(SE)	p-value
sex	0.0361	0.121	0.728	−0.9609	0.2224	1.56e–05
lake	−0.0424	0.170	0.680	−0.5199	0.2122	0.01430
treatment	0.1588	2.398	0.121	1.0765	0.3935	0.00623

The first three columns (“Proportional hazards “) provide model diagnostics of the employed Cox proportional-hazards regression, i.e. they test the proportional hazards assumption for the model fit. The non-significant p-values of the statistics indicate that the assumption is not violated. The fourth column (coef [δ]) provides the model estimates for the three coefficients and the fifth column the respective standard errors (SE). All tested covariates had a significant effect (p-values in the sixth column).

## Discussion

We observed considerable differences in UV behavioural response, mortality, and pigmentation plasticity between lake populations and sexes, despite identical laboratory conditions and the exclusion of putative maternal effects. In order to explain these differences we will extend the well-studied trade-off triangle between UV stress, carotenoid pigmentation, and fish predation, e.g. [20], [38], and discuss the observed complexity of responses as a combination of lake-specific biotic and abiotic factors. Our argumentation is built on the following assumptions: (1) Omnivorous calanoid copepods benefit from time spend in surface waters due to access to algal food, which in turn increases survival and fitness [45], [46]. (2) An increased surface time diminishes, however, survival due to higher UV stress and predation risk by fishes [47], [48]. (3) Under low predation pressure this trade-off can be shifted toward a prolonged surface time by employing protective carotenoid pigmentation [16], [49].

### Lac Pavin

The LP population exhibits a distinct sexual dimorphism in carotenoid pigmentation (Fig. 1). Moreover, males were able to increase astaxanthin content after prolonged UV exposure whereas females remained translucent (Fig. 2). Despite this dimorphism, both sexes responded uniformly to UV cues with a pronounced downward movement (Fig. 3A, B). The question arises as to why both sexes entertain considerable differences in protective carotenoid pigmentation.

The stronger downward movement of females after UV cue (Fig. 3B) is a direct consequence of the differential distribution of sexes under white light: while males were uniformly distributed in the water column (β_2_ in Table 1), females occupied predominantly the upper compartment (β_4_ in Table 1; Fig. 3C). As this female preference was not observable in the male-free containers, we interpret this behaviour as a consequence of sexual conflict. This argument is further supported by frequent observations of female escape jumps following male copulation trials and the regular occurrence of multiple spermatophores being attached to individuals (pers. observ., 2011). The spermatophores stick to the exoskeleton and decrease manoeuvrability while simultaneously increasing mortality [32]. Females are hence under considerable selective pressure to limit mating encounters.

The ability of LP males to plastically change carotenoid pigmentation under UV stress (Fig. 2) and the fact that they exhibited the lowest mortality during the experiment (Fig. 4A) suggest an adaptation to surface water habitats. We argue that males maximize sexual encounters–and in their case fitness–by remaining in nutrient-rich but UV affected surface waters. Such a male strategy was also reported from other freshwater copepod species [45], [50]. The seemingly contradictory preference of LP males for the lower compartment of the water column under UV light (β_1_ in Table 1) does hence not indicate a preference for deeper, UV-unaffected waters but results most likely from the artificial depth limitation of the containers in the experiment.

We argue that LP females, in contrast, maximize fitness by moving into deeper water during daytime, thus simultaneously escaping excessive mating trials and UV radiation, both of which substantially increased their mortality compared to LP males and to the negative control (Fig. 4A, Table 2). Consequently, LP females emerge probably only for nocturnal feeding (a similar behaviour is discussed for marine copepods; [51], [52]). Additionally, the minimization of surface time would decrease fish predation pressure to which females are more vulnerable than males due to their larger sizes [53]. This line of arguments offers a straightforward explanation for the observed sex-specific UV response in LP: Whereas the restriction of females to deeper waters during daytime renders carotenoid pigmentation unnecessary, it still demands the maintenance of the UV sensitive sensory system. Consequently, females have lost their ability to encounter UV threat with carotenoid pigmentation (Fig. 2, Fig. 4A), yet strongly respond to UV cue with a downward movement (Fig. 3B). Males on the other hand are adapted to plastically respond to UV radiation at the water surface (Fig. 4A) in order to maximize mating encounters and thus their fitness.

### Lac de Montcineyre

In contrast to LP specimens, the comparison of LM copepod distributions under white- versus UV light revealed no downward movement following UV cue (Fig. 3D, E). On the contrary, males tended to react with a weak upward movement to UV stimulus (note the shift on the posterior distribution to the left in Fig. 3D), whereas the distribution of females did not change (β_7_ and β_8_, Fig. 3E). There is, however, a clear separation of the sexes under white light (Fig. 3F). Moreover, we also observed multiple spermatophore transfers and female escape jumps in the LM population, indicating a similar male-avoiding strategy of LM females. Yet despite the apparent sexual conflict in the LM population, males were translucent and did not accumulate carotenoids during UV exposure (Fig. 2). If pigmentation constitutes a prerequisite for males to maximize their fitness by remaining in surface waters (as in LP), the question arises as to why there is no pigmentation dimorphism in LM.

The lack of a plastic pigmentation response in LM males to UV stress indicates a substantially different selective regime in this lake compared to LP. Differences in mortality rates between males from LM and LP indicate a higher tolerance of UV radiation in LP males (Fig. 4A, B) and, by extension, comparatively low UV stress in LM. One potential reason for this is a lake-specific difference in UV penetration depth, which is positively correlated with water clarity [12], [54], [55]. The overall water clarity in LM appears to be lower than in LP (pers. observ., 2011), most likely due to runoff from adjacent agricultural areas. In contrast, LP is protected from such runoff by a wooden relief barrier around the lake (pers. observ., 2011, Fig. 1). Moreover, the macrophyte vegetation in the LM littoral may offer additional shelter from UV radiation (*sensu* Schneider et al. [56]).

Whereas these considerations offer an explanation as to the difference in physiological response between LM and LP populations, they do not explain the striking behavioural difference (Fig. 3A, B versus 3D, E). Sexual conflict would force LM females to minimize male encounter by descending into deeper waters during daytime. However, this does not seem to happen (Fig. 3E). A potential cause for this behaviour is a planktivorous predator, which is present in LM but not in LP: the larva of the dipteran genus *Chaoborus* Lichtenstein, 1800 (pers. observ., 2011; Ringelberg, pers. comm., 2010). *Chaoborus* responds to UV cue by moving into deeper waters, therewith escaping UV stress and fish predation [57]. Copepods, in turn, actively avoid *Chaoborus* predation by moving closer to the surface during daytime [53], [58]. The observed weak positive UV phototaxis in LM but not in LP males may thus be explained as an adaptation to a LM specific predation risk: UV radiation serves as proxy for dawn and induces an upward swimming of copepods from the descending *Chaoborus* larvae. For LM females this poses a dilemma: In order to escape *Chaoborus* predation they must leave the deeper waters during daytime while sexual conflict requires them to avoid surface waters. A possible solution is a UV induced separation of sexes in a horizontal plane, as reported for an Austrian *A. denticornis* population and other copepod species [59], [60]. Thereby both sexes inhabit different lake parts during daytime without descending into *Chaoborus* infested deep water.

## Conclusions

As all other described European populations of *A. denticornis* are pigmented, it appears reasonable to assume that the plastic red LP morph is ancestral and the translucent LM morph derived. Hence, the slightly higher carotenoid concentration in LM males as compared to females of both lakes (Fig. 2) may constitute a remnant of a once stronger, ancestral male pigmentation – similar to present day LP males. The presence of the *Chaoborus* larvae in LM, however, might have forced the copepods to undergo considerable physiological and behavioural changes. Selection towards these changes must have been strong as–in contrast to LP males–any plasticity for carotenoid concentration in LM males seems to be lost (Fig. 2). Despite the absence of predators in our experimental system for over 12 generations, LM males did neither respond to UV stress by accumulating carotenoids nor did either sex attempt to escape radiation by a downward movement. We thus consider both traits in the LM population as fixed adaptations to a different ecological niche and the loss of plasticity in carotenoid pigmentation as genetic assimilation following an altered selection regime in LM. Our study hence strongly argues in favour of the initially stated hypothesis that PP will be higher when selection pressure is moderate and will decrease or even be lost under stronger pressure. In light of the presumed age of both lakes of ∼6000 years [23], it is reasonable to assume that the here reported loss of PP happened within this timeframe.

The present study furthermore illustrates how a change in a one parameter (i.e., the addition of a new predator) affects other traits of the population by disturbing a fine-tuned trade-off system comprising sexual conflict, UV stress, carotenoid pigmentation, and predation. In light of the fixed physiological and behavioural traits in our LM population, it is conceivable that selection against immigrants or interlacustrine hybrids with intermediate (i.e., putatively ill-adapted) phenotypes may reinforce an on-going speciation process [2], [61].
